# The *E*. *coli* dicarboxylic acid transporters DauA act as a signal transducer by interacting with the DctA uptake system

**DOI:** 10.1038/s41598-017-16578-w

**Published:** 2017-11-27

**Authors:** Eleni Karinou, Paul A. Hoskisson, Alexander Strecker, Gottfried Unden, Arnaud Javelle

**Affiliations:** 1 Division of Molecular Microbiology, College of Life Sciences, University of Dundee, Dundee, DD1 5EH UK; 2 Strathclyde Institute of Pharmacy and Biomedical Sciences, University of Strathclyde, 161 Cathedral Street, Glasgow, G4 0RE UK; 3 Institute for Microbiology and Wine Research, Johannes Gutenberg-University, Mainz, Germany; 4 Present Address: Section of Microbiology and MRC Centre for Molecular Bacteriology and Infection, Imperial College London, London, United Kingdom

## Abstract

The Slc26A/SulP family of ions transporter is ubiquitous and widpsread in all kingdon of life. In *E*. *coli*, we have demonstrated that the Slc26 protein DauA is a C_4_-dicarboxilic acids (C_4_-diC) transporter active at acidic pH. The main C_4_-diC transporter active at pH7 is DctA and is induced by C_4_-diC via the DcuS/R two component system. DctA interacts with DcuS, the membrane embedded histidine kinase, to transfers DcuS to the responsive state, i.e. in the absence of DctA, DcuS is permanently “on”, but its activity is C_4_-diC-dependent when in complex with DctA. Using phenotypic characterization, transport assays and protein expression studies, we show that at pH7 full DctA production depends on the presence of DauA. A Bacterial Two Hybrid system indicates that DauA and the sensor complex DctA/DcuS physically interact at the membrane. Pull down experiments completed by co-purification study prove that DauA and DctA interact physically at the membrane. These data open a completely new aspect of the C_4_-diC metabolism in *E*. *coli* and reveals how the bacterial Slc26A uptake systems participate in multiple cellular functions. This constitutes a new example of a bacterial transporter that acts as a processor in a transduction pathway.

## Introduction

Under aerobic conditions, *Escherichia coli* can oxidise C_4_-dicarboxylic acids (C_4_-diC, succinate, fumarate, malate, tartrate, aspartate and the aromatic monocarboxylate orotate) and use them as sole carbon and energy sources^1^. At neutral pH, the main C_4_-diC importer is DctA (TC 2.A.23.1.7), from the dicarboxylate/amino acid:cation symporter (DAACS) family of carriers. DctA has a wide repertoire of substrates (succinate, fumarate, malate, aspartate, tartrate, orotate)^2^. It has been shown that a *dctA* mutant retains the ability to grow on succinate as the sole carbon source under acidic conditions, suggesting there is another succinate transporter encoded in the genome of *E*. *coli* ^3^. We identifed DauA (previously named YchM, TC 2.A.53.3.11) as the main transporter for C_4_-diC (succinate/fumarate/aspartate) active under acidic conditions^4^ and in subsequent work, DauA from *Deinococcus geothermalis* was reported to act as a proton/fumarate symporter^5^, with the *Salmonella enterica* serovar Typhimurium homologue displaying fumarate binding activity^6^. DauA belongs to the Solute Carrier 26 (Slc26) and sulphate transporter (SulP) family of ion transporters which is a superfamily of transporter proteins conserved from bacteria to man^7^. Proteins within the Slc26/SulP family are involved in various biological functions, transporting a broad spectrum of substrate, from ions to carboxylic acids^8–10^. At least 10 Slc26 transporter have been identify in human, playing critical role in various cellular function and are medically relevant, acting as tumor suppressors or being involved diseases such as congenital chloride diarrhea, diastrophic dysplasia, Pendred syndrome and nonsyndromic deafness^9^. SulP proteins in fungi and plants are uptake system for sulfate^11,12^. Members of the Slc26/SulP family are diverse in terms of substrate specificity and mechanism of transport but share a common protein structure. They contain an integral membrane domain containing two inverted repeats of seven transmembrane segments, resembling the fold of the uracil transporter, UraA, and a cytoplasmic Sulfate Transporter and Anti-Sigma Factor Antagonist (STAS) domain^5,13,14^. Although the function of the STAS domain has yet to be fully elucidated, it was thought to be important for targeting the proteins to the membrane. Recent work replaced the bacterial Slc26A transporters STAS domains with the green fluorescent protein (GFP) and demonstrated that the STAS domain does not play a direct role in protein targeting in bacteria, but rather it is required for protein stabilization and functionality^14–17^.

We have demonstrated in a previous study that in *E*. *coli*, DauA and DctA were the main aerobic C_4_-diC transporters under acidic and neutral conditions, respectively^4^. At pH7, the expression of *dctA* is induced by external C_4_-diC via the DcuS-DcuR two-component regulatory system where DcuS is the membrane embedded histidine kinase and DcuR the DNA binding response regulator^18^. It has also been reported that DctA, via a cytoplasmic amphipatic helix (8b), interacts with the cytoplasmic PAS (Per, Arnt, Sim) domain of the membrane embedded sensor kinase DcuS^19^. It was proposed that DctA acts as a co-sensor in the DctA/DcuS complex^19^, however more recent studies indicates that DctA controls the functional state of DcuS such that in the absence of DctA, DcuS is permanently “on” but in the presence of DctA, the DcuS activity is dependent on the presence of external C_4_-diC^20^. Under anaerobic condition, the transporter DcuB takes over the role of DctA and interacts with DcuS^21^. We have shown that in the absence of DauA, at neutral pH, DctA expression and activity was reduced, indicating that DauA does not only transport C_4_-diC at acidic pH but is also involved in the regulation of carboxylic acids metabolism^4^. Hence, the present study aimed to define the role of DauA in C_4_-diC metabolism at neutral pH. Phenotypic characterization, *in vivo* assays of fumarate transport and studies of proteins expression provide compelling evidence that high-level of DctA production and activity depends on the presence of DauA at pH7. *In vivo* studies using a Bacterial Two Hybrid system (BTH) and fluorescence microscopy indicates that DauA and the sensor complex DctA/DcuS interact physically at the membrane to facilitate the cross-talk between the two systems. The complex formed by DauA and DctA/DcuS was further characterized biochemically. Surprisingly, co-purification/mass spectrometry analysis suggested that DauA interacts with DctA but not DcuS. This finding was confirmed *in vitro* by pull-down experiments. Finally, by co-expressing various truncated proteins in the BTH system, we show that DctA and DauA interact via their transmembrane domain and not their helix 8b and STAS domains respectively. This result reveals a completely new aspects of C_4_-diC metabolism in *E*. *coli* and suggest a multifunctional role of DauA in *E*. *coli* physiology, possibly linking fatty acid and C_4_-diC metabolism.

## Results

### DauA is necessary for activity/production of DctA at pH7

**Figure 1:**
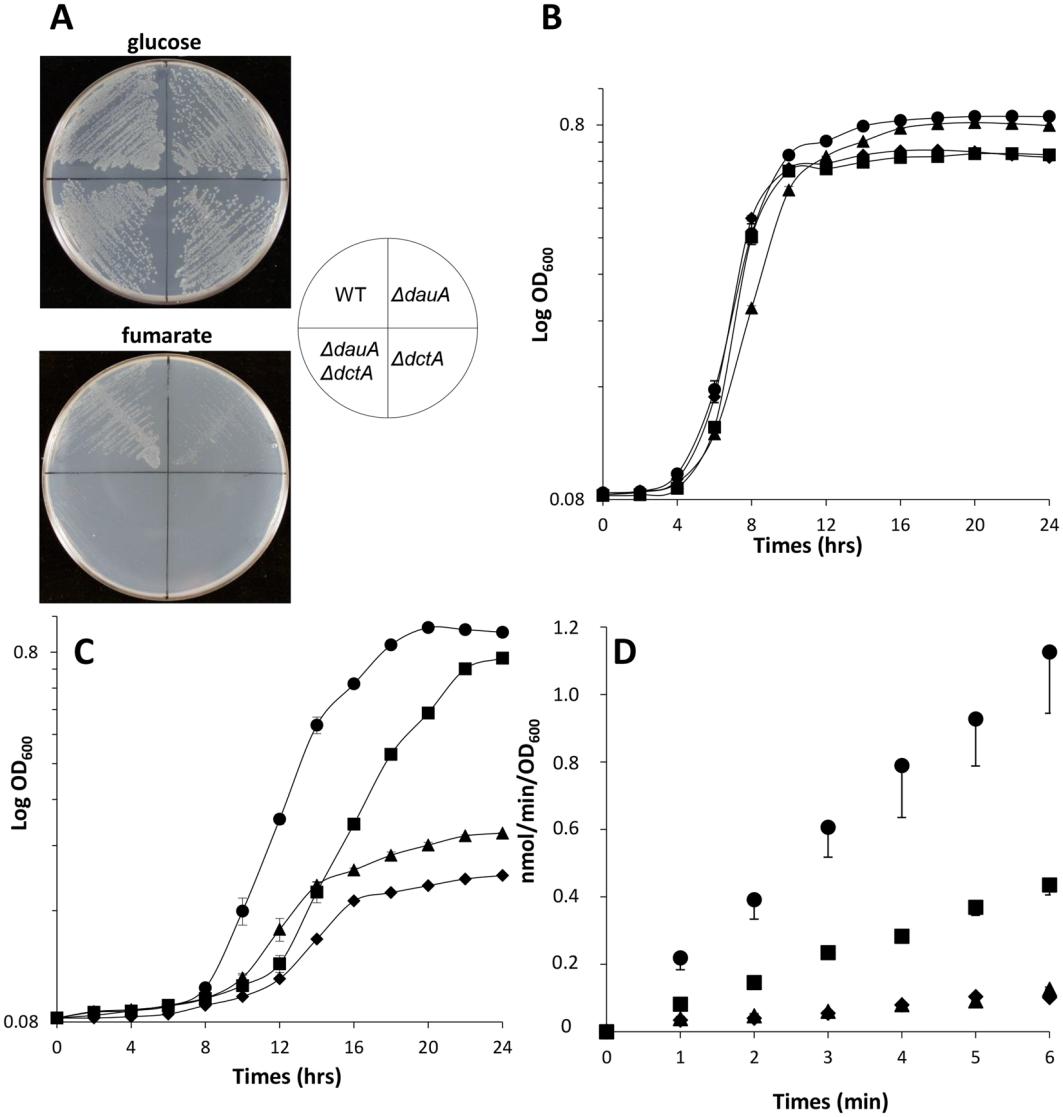
The production/activity of DctA depend on the presence of DauA. (**A**) Growth of the isogenic wild-type and Δ*dctA*, Δ*dauA* and Δ*dctA*/Δ*dauA* strains on MOPS minimal medium (pH 7) containing 50 mM of fumarate or glucose as sole carbon source. The plates were incubated for 24 hrs at 37 °C. **(B)** The wild-type (●),Δ*dctA* (▲),Δ*dauA* (■) and Δ*dctA/*Δ*dauA* (♦) strains grew in eM9 minimal medium at pH 7 containing 50 mM glucose as sole carbon source. Growth at 600 nm were monitored using microplates. All results are the averages of at least 4 independent test series. Error bars represent the standard deviation. **(C)** Same than B but the medium contained 50 mM fumarate as sole carbon source. **(D)** Time dependence of [^14^C]-fumarate accumulation in BW25113 (wild-type; ●), EK1 (Δ*dauA*; ■), EK2 (Δ*dctA*; ▲) and EK3 (Δ*dctA/*Δ*dauA*; ♦) strains. Measurements done after the cells were grew for 6 hrs in M9 containing 50 mM fumarate as the sole carbon source at pH 7. All results are the averages of at least 6 independent test series. Error bars represent the standard deviation.

We have previously observed that aΔ*dauA* mutant is defective in growth at pH7 on minimal medium where succinate is the sole carbon source^4^. This led us to hypothesize that DauA plays a role in the regulation of C_4_-diC metabolism at neutral pH. To test this hypothesis, we examined the monitored aerobic growth of the isogenic wild type and Δ*dauA*, Δ*dctA* and Δ*dauA/*Δ*dctA* strains, on plates with minimal medium (pH7) containing 50 mM of either glucose or fumarate as the sole carbon source (Fig. 1A) for 24 hr at 37 °C. Although the wild type strain did not present any growth defect, the dauA mutant grew less well and the other two strains did not grow. As a control, it has to be noted that all four strains grew equally well in the presence of glucose (Fig. 1A). In order to quantify these phenotypes, we compared the growth in liquid eM9 medium of the same four strains at pH7, supplemented with 50 mM glucose (Fig. 1B) or 50 mM fumarate (Fig. 1C) as sole carbon source. All four strain grew equally well on glucose with a specific growth rate (μ) of = 0.13 h^−1^ for the wild-type, Δ*dauA*, Δ*dauA/*Δ*dctA* and 0.12 h^−1^ for Δ*dctA* respectively (Fig. 1B). On fumarate as sole carbon source, the Δ*dauA* strain did not grow quite as well (μ) = 0.04 h^−1^) as the wild-type strain (μ) = 0.07 h^−1^), whilst the Δ*dctA* and the double mutant strains showed equally poorest growth (μ) = 0.01 h^−1^; Fig. 1C). Together these data confirm that DctA is the main fumarate transporter at pH 7 and suggests that DauA is essential for full DctA activity/expression.

To understand whether DauA is essential for DctA activity or production, we measured the *in vivo* [^14^C]-fumarate uptake in the isogenic wild type and Δ*dauA*, Δ*dctA* and Δ*dauA/*Δ*dctA* strains. [^14^C]-fumarate accumulation were measured by filtration after addition of 40 μM [^14^C]-fumarate for six minutes. The cells trapped on the filter were washed and the level of radioactivity measured. These [14C] accumulation was measured performed after induction of the cells for 6 hr in fumarate at pH 7. The [^14^C]-fumarate uptake activity was linear over 6 mins for all of the strains tested (Fig. 1D), with the uptake activity for the wild-type strain being 17.7 ± 2.8 nmol/min/OD_600_ and the Δ*dauA* strain exhibiting 2.6-fold lower rates of uptake (6.7 ± 1.0 nmol/min/OD_600_). A negligible accumulation was measured for the Δ*dctA* and Δ*dauA/*Δ*dctA* cells.

To assess whether the reduced DctA transport activity observed in the absence of DauA correlated with a decrease of DctA production, we examine the amount of DauA and DctA present when fumarate was supplied as the sole carbon source in a wild-type, Δ*dctA* and Δ*dauA* background respectively.

In order to measure the proteins production, we introduced, in the chromosome, an His tag fused with the C-terminal and of *dauA* in the wild-type and Δ*dctA* backgrounds and a fused with the C-terminal end of *dctA* in the wild-type and Δ*dauA* backgrounds.

**Figure 2:**
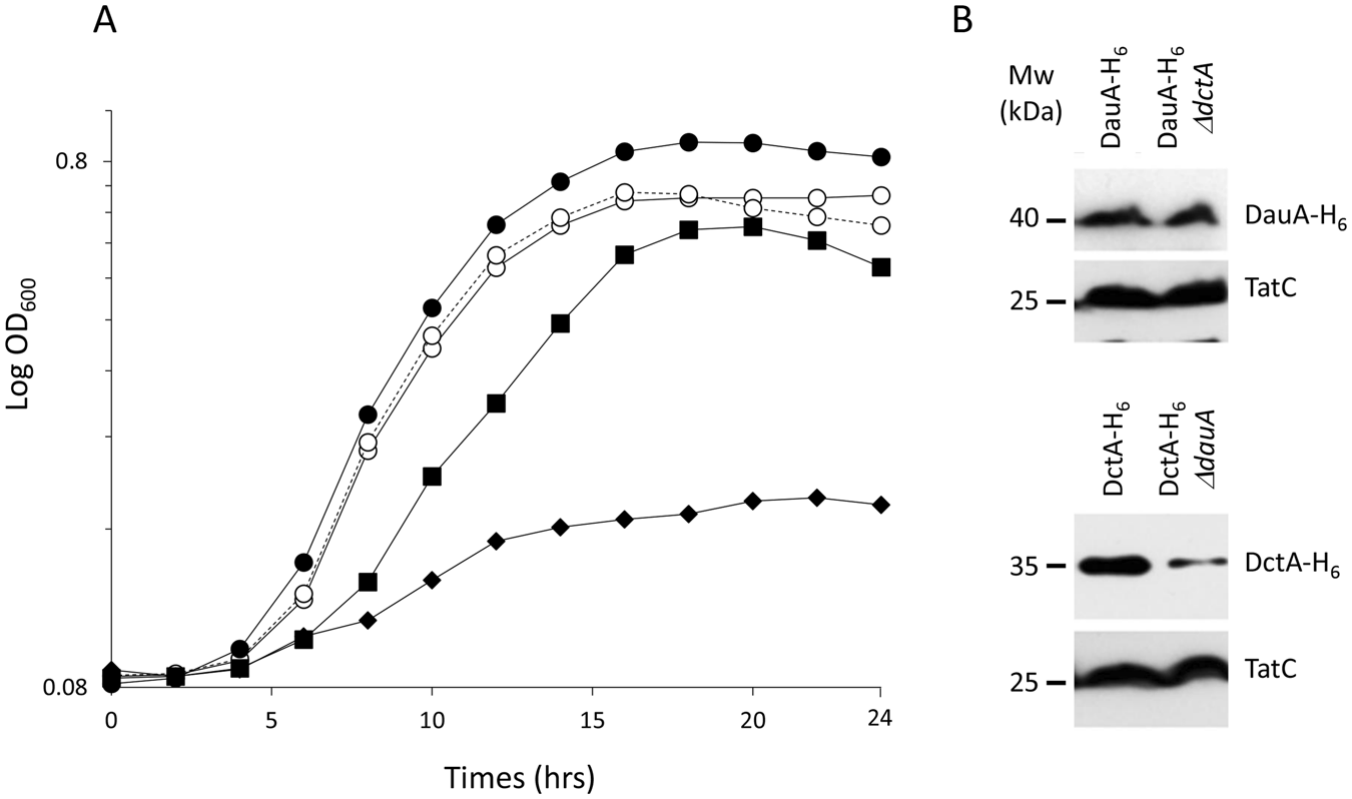
The presence of DauA is necessary for optimal DctA production. **(A)** The wild-type (●),DauA-H_6_ (○), DctA-H_6_ (dashed line), DauA-H_6_Δ*dctA* (♦) and DctA-H_6_Δ*dauA* (■) strains were grown in eM9 minimal medium at pH 7 containing 50 mM fumarate as sole carbon source and the curves at 600 nm were recorded using microplates. All results are the averages of at least 4 independent test series. **(B)** Membrane fractions were prepared from DctA-H_6_, DctA-H_6_Δ*dauA*, DauA-H_6_, DauA-H_6_Δ*dctA* after growth for 6 hrs in M9 minimal medium supplemented with 50 mM fumarate as sole carbon source at pH 7. Samples were subjected to SDS-PAGE, followed by Western blotting with an anti-His antibody. The same membranes were subsequently stripped and re-hybridized with a polyclonal anti-TatC antibody as a control.

The presence of this epitope tag did not affect both proteins function as the growth of the strains expressing Strains DauA-H_6_ (μ) = 0.059 h^−1^) and DctA-H_6_ (μ) = 0.063 h^−1^) on minimal medium containing fumarate as sole carbon source was similar to the growth to the strains producing the native forms of DauA and DctA (μ) = 0.071 h^−1^), and likewise strains DauA-H_6_, Δ*dctA* (μ) = 0.007 h^−1^) and DctA-H_6_, Δ*dauA* (μ) = 0.043 h^−1^) present a similar groteh compare to the strains producing untagged proteins (Figs 1C, 2A). To measure the presence of DctA in cells growing on fumarate, we grew strains overnight in M9 medium supplemented with glucose as the carbon source, washed then two time and subsequently cultured for 6 hrs on M9 minimal medium containing fumarate. We examined for the presence of DctA and DauA in the membrane fractions by Western immunoblotting using anti-His antibodies, with the constitutively expressed TatC used as a membrane protein loading control. DctA-H_6_ and DauA-H_6_ where only detected in the membrane, indicating that the proteins were correctly targeted to the membrane (data not shown). The production of DauA-H6 was equivalent in the wild type and Δ*dctA* strains (Fig. 2B). It was also apparent that less DctA-H_6_ was produced in the DctA-H_6_Δ*dauA* strain, suggesting that the presence of DauA is necessary for optimal DctA production (Fig. 2B). It also confirms our hypothesis in light of the data for the transport assay and the DauA-dependent phenotype observed in a Δ*dauA* strain in minimal medium with fumarate as sole carbon source.

### DauA interacts *in vivo* with the DctA/DcuS complex

**Figure 3:**
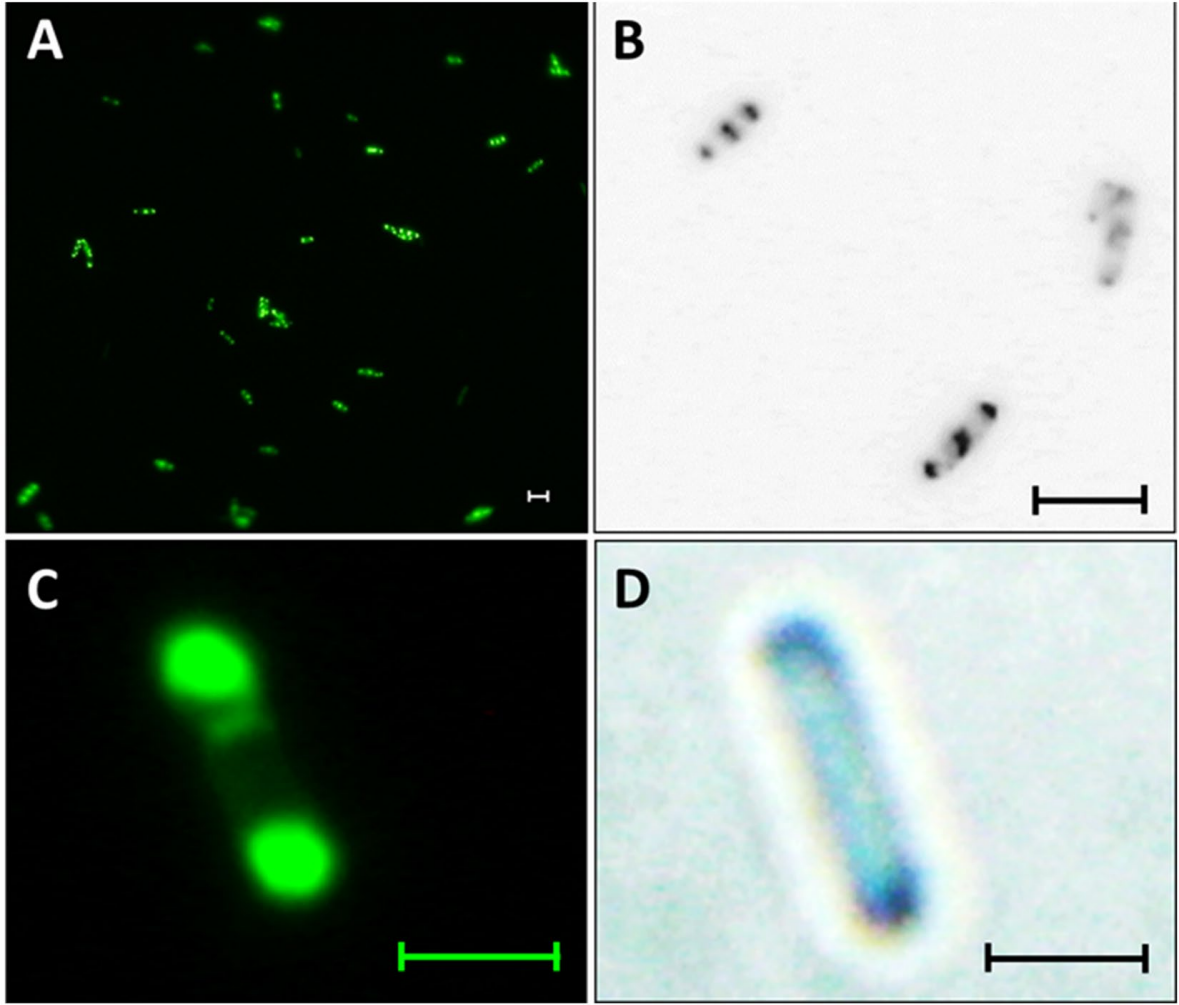
Polar localization of mYFP-DauA in *E*. *coli* EK1 (*∆dauA* with plasmid pMW2010 encoding *his6-mYFP-dauA)*. The bacteria were grown in the presence of 10 µM (C, D) or 50 µM (**A**,**B**) arabinose and analyzed by fluorescence microscopy, pictures B presented in black and white, blue coloring. (**A**) represents an overview, C and D single cells. The bars correspond to 5 µM (**A**), 3.5 µM (B), and 2.5 µM (**C**,**D**).

It is known that DctA forms a complex at the membrane with the sensor kinase DcuS, the complex being localized at the poles of the cell. Hence we look at the localization of DauA to assess if itis also localize at the cellular pole. The subcellular localization of DauA was tested by *in vivo* fluorescence microscopy of a N-terminal fusion of DauA to monomeric mYFP. Expression of *mYFP-dauA* is under the control of the *ara* promoter. Bacteria grown with 10 µM or 50 µM arabinose showed fluorescence, but not those grown with 0 µM Ara. From bacteria induced with 10 µM or 50 µM arabinose 91 and 76 cells were analyzed for fluorescence (Fig. 3A). The bacteria were selected from 5 random sections each, and all bacteria within these sections were analyzed. From the bacteria grown with 10 µM arabinose 21% (19 of 91 cells) showed fluorescence, from the 50 µM arabinose grown 39% (30 of 76 cells). The green fluorescent spots of the mYFP fusion protein from all bacteria with fluorescence had polar localization (Fig. 3B–D), even in the bacteria with very low induction (10 µM arabinose). Polar localization depends on the fusion of mYFP to DauA, whereas free YFP is randomly distributed in the cellular cytoplasm. The polar localization is specific for DauA (Fig. 3) since membrane proteins with random distribution in the membrane like the Tar mutant Tar(1–331) show membraneous, but no polar localization^37^. The amounts of mYFP-DauA that were present in the bacteria with polar localization were estimated by immunoblotting (Fig. S1). In the experiment the levels of mYFP-DauA were compared to chromosomally encoded DauA-His6. Expression of the chromosomal *dauA-his6* is under the control of the *dauA* promoter which causes expression of DauA-His6 at wild-type levels. The levels of YFP-DauA in the bacteria grown with 10 µM arabinose are very similar to those of *E*. *coli* expressing His6-DauA at pH 5 under succinate induction. Therefore the polar localization of mYFP-DauA which is shown in Fig. 3 is not due to artificial over-production or aggregation artifacts of the protein. This observation is in agreement with the model put forward that DauA interacts with the (polar) DctA/DcuS complex^4^.

**Figure 4:**
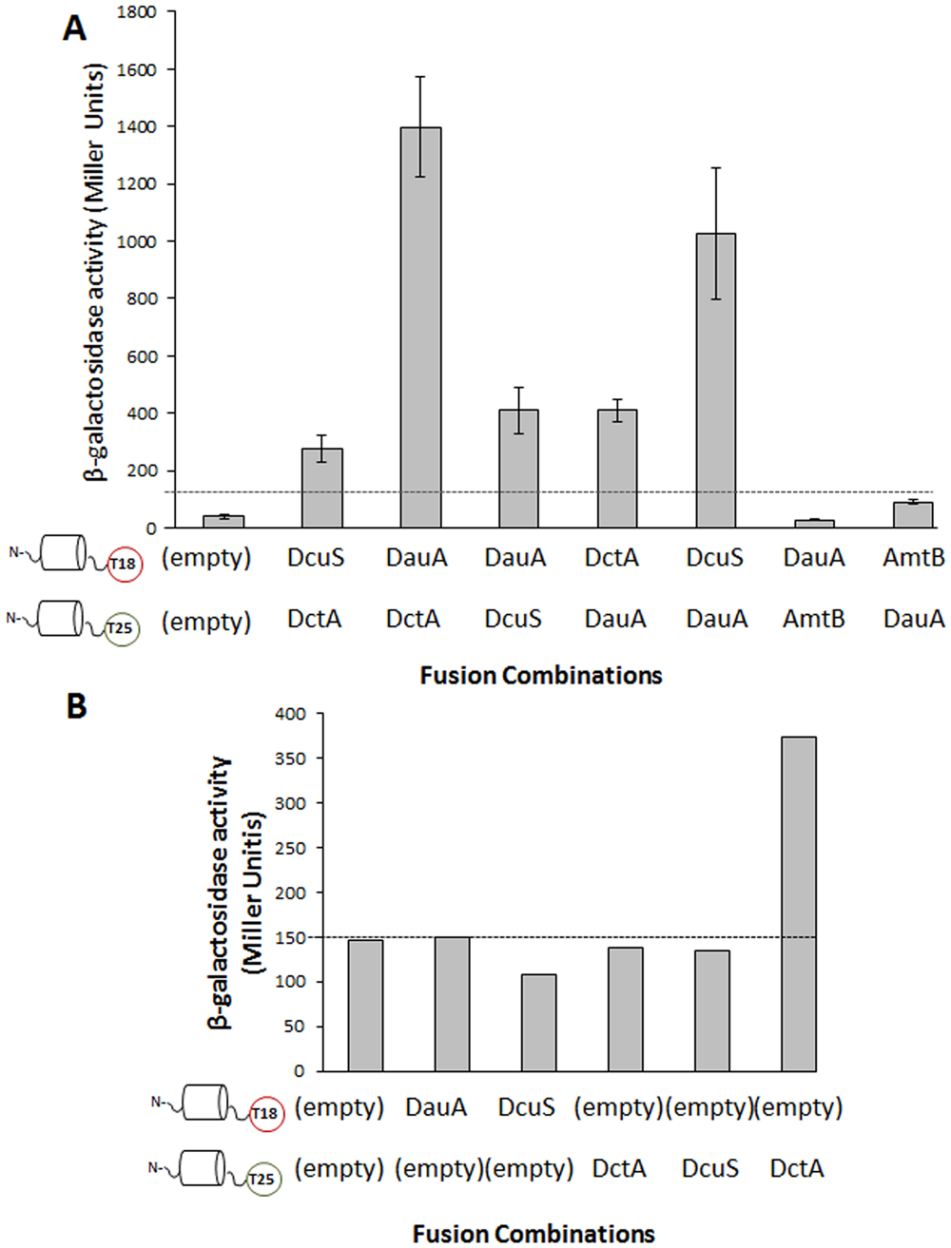
Interaction of DauA with DctA/DcuS. **(A)** β-galactosidase activity was used to quantify the BTH results. BTH101 (*cyaA* ^*−*^ reporter strain) was co-transformed with the respective plasmid pairs. The activity measure for the pair DctA-T25/DcuS-18 was used as a positive control. All the results are the average of at least five independent biological repeats. Error bars represent standard error of the mean. **(B)** The background activity was measured by non-interacting pairs and was determined below 150 Miller units. The data presented are representative of one experiment.

Secondly, To identify any potential interaction between DauA and DctA and/or DcuS, we used a Bacterial Two Hybrid (BTH) system approach, utilising the functional reconstitution of adenylate cyclase (CyaA) from the separate T18 and T25 domains in an *E*.*coli cyaA* ^*−*^ reporter strain^22^. In the BTH system, the two candidate proteins to be tested for interaction can be N- or C-terminal fused with the T18 or T25 domain of CyaA. Previous work in our group has shown that production of DauA may be affected by altering the N-terminus^14^. To avoid this we used the pUT18 and pKNT25 plasmids that result in C-terminal fusions of DauA, DctA and DcuS with the T18 and T25 domain. In this assay, we used the known interacting pair DctA-DcuS as a positive control (Fig. 4A – 252 miller units). The background activity (negative control) was measured by transforming the cell with non-interacting pairs and determined as being bellow 150 millers units (Fig. 4B). Interestingly, the activity measured for the pairs DauA-T18/DctA-T25 (1202 Miller units) and DcuS-T18/DauA-T25 (960 Miller units) were 5 and 4 times higher respectively than the positive control DcuS-T18/DctA-T25 (252 Miller units), and more than 8 times higher than the background activity (below 150 Miller units). The reciprocal pairings of DauA-T18/DcuS-T25 (408 Miller units) and DctA-T18/DauA-T25 (412 Miller units) also showed high β-galactosidase activity, suggesting there is interaction between DauA and DctA and DcuS. One concern when expressing membrane proteins from the high copy plasmid pUT18 is that they might saturate the cell membrane resulting in non-specific interactions. To verify that interactions between DauA and DctA and/or DcuS were specific, we tested the interaction between DauA and the membrane protein, AmtB. AmtB is an ammonium transporter which is not known to interact with any components of the C_4_-diC acid metabolism. The β-galactosidase activity measured when DauA and AmtB were co-expressed in the reporter strain *cyaA* ^*−*^ was below the background level (150 Miller units), suggesting the observed DauA interactions with DctA and DcuS are indeed specific.

### DauA interacts *in vitro* with DctA but not DcuS

The data above suggest that DauA may physically interact with both DctA and DcuS. The explanation for this results is certainly due to the fact that the BTH reporter strain *cyaA* ^*-*^ expresses chromosomally encoded DctA and DcuS. It is possible that the chromosomally encoded proteins interact with the T18/T25 chimeric proteins encoded by the plasmid. This means that DauA fused with the fragment T18 or T25 interact with the chromosomally encoded DctA which would bring DauA-(T18/T25) in close proximity with DcuS-(T18/T25) and thus gives positive result with the DauA/DcuS pair. The inverse may also explain the positive result i.e. DauA-(T18/T25) interacting with the chromosomally encoded DcuS that give a positive result with the DauA/DctA pair. Therefore, in order to distinguish whether DauA interact directly with DctA or DcuS, we sought to confirm the interaction biochemically. Through the expression of DauA at the native levels from the chromosome, we looked for proteins which co-purified with DauA, using DauA-H_6_ and WT cells as a control. The cells were grown overnight in LB supplemented with fumarate as an inducer of DctA and DauA was purified initially by IMAC. The elution fractions of two biological replicates were analyzed by nano-liquid chromatography on-line coupled to mass spectrometry (nLC-MS/MS). Proteins identified in the non-tagged strain grown under the same conditions were considered non-specific and they were subtracted from the protein list. Proteins that were present in both biological replicates were considered as potential DauA interacting partners (Table 1). Firstly, DctA was identified in the list, but not DcuS supporting our hypothesis that DauA interacts with DctA. The low sequence coverage and small number of unique peptide detected for DctA and DauA reflect the difficulty in analysing integral membrane proteins by nLC-MS/MS.

**Figure 5:**
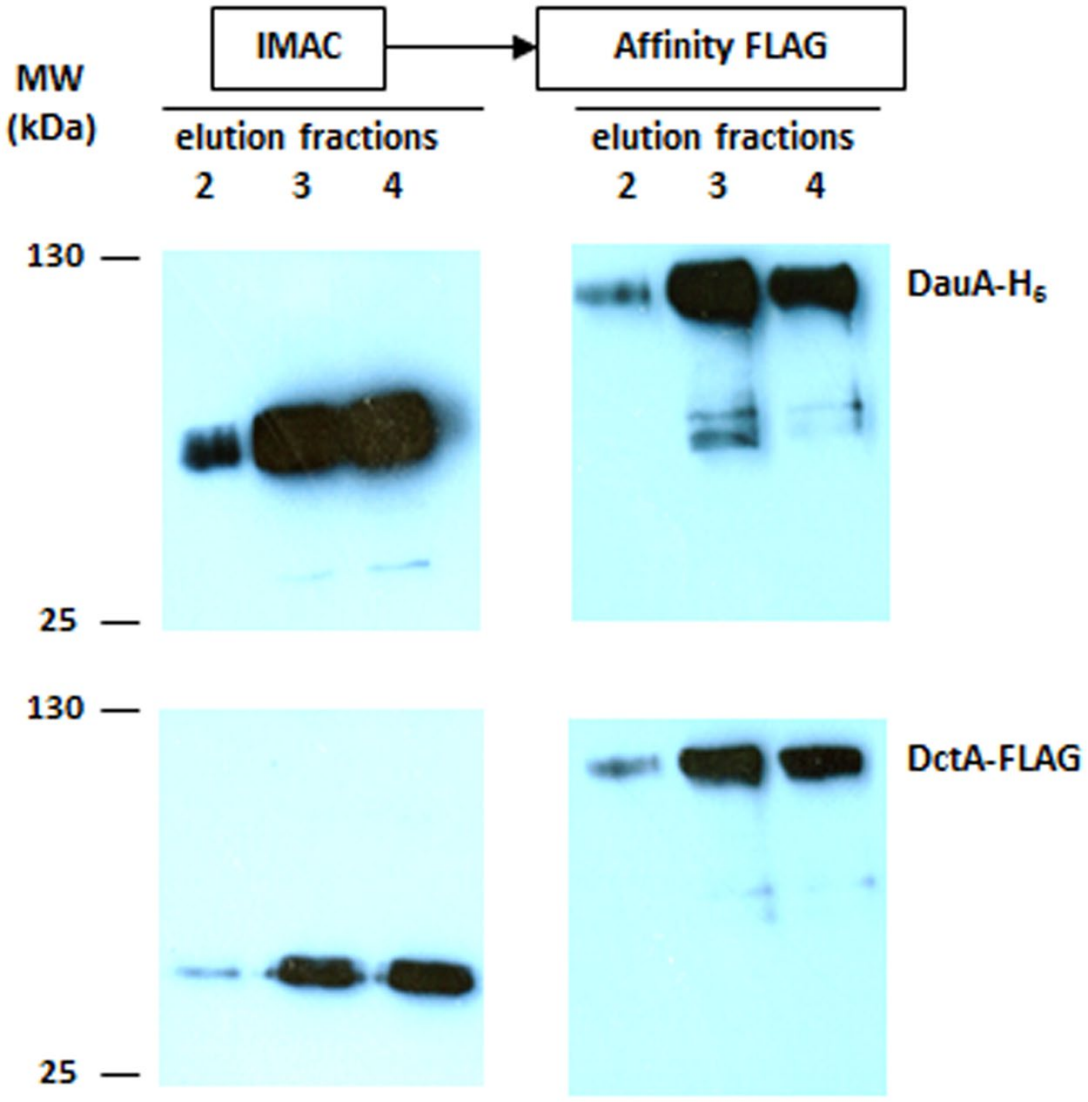
DauA and DctA form a complex. Chromosomally encoded C-terminally His-tagged DauA was purified by IMAC. Elution fractions were analyzed by Western immunoblotting using an anti-His antibody (top panel) and anti-FLAG antibody (bottom panel). The IMAC elution fractions were re-purified by FLAG affinity chromatography and analysed by Western blotting.

To confirm that DauA and DctA are interacting *in vitro*, a strain harboring a chromosomally encoded His-tagged version of DauA and a chromosomally encoded FLAG-tagged version of DctA were used. The cells were grown overnight in LB supplemented with fumarate as an inducer for DctA expression and DauA was purified by immobilized metal ion affinity chromatography (IMAC). The eluted fractions were further purified by FLAG affinity chromatography with the presence of DauA and/or DctA in the fractions monitored by western blotting using anti-His or anti-FLAG antibodies. We hypothesized that if we re-purify the IMAC eluted fractions using an anti-FLAG matrix we should be still able to detect both DauA and DctA if these proteins interact *in vivo*. Initial IMAC purification detected DauA in the eluted fractions (Fig. 5). The membrane was subsequently stripped and reblotted using anti-FLAG antibody, revealing the presence of DctA in the same elution fractions (Fig. 5). The same fractions where analyzed for the presence of DcuS using a specific anti-DcuS antibody however no signal was detected, indicating that DauA does not interact with DcuS directly. Next, these elution fractions were purified using an anti-FLAG matrix. The western blot analysis of separate membranes for each antibody (His and FLAG) showed that DctA co-purified with DauA. We observed an apparent increase in molecular mass for both proteins following the second purification and is likely due to aggregation because of the acidic conditions used to elute the protein from the anti-FLAG matrix. Taken together, these data from the co-purification/mass spectrometry analysis and pull-down experiment confirmed that DauA and DctA interact physically *in vivo*.

### DauA and DctA interact via their transmembrane domain

**Figure 6:**
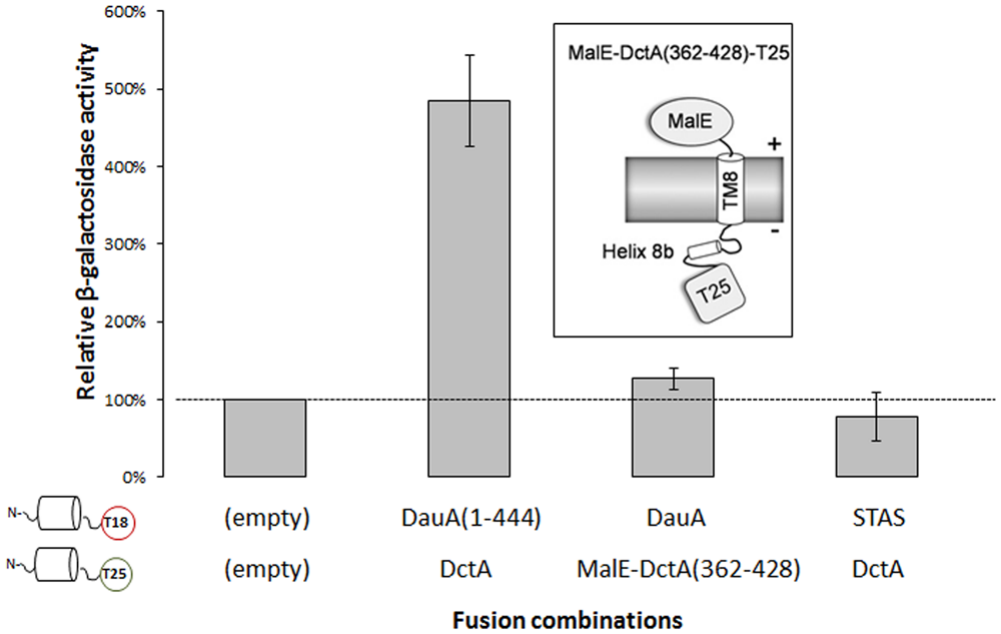
DauA sites required for interaction with DctA. β-galactosidase activity was used to quantify interaction. BTH101 cells co-transformed with the respective plasmid pairs. The activity was determined after growth in LB supplemented with the appropriate antibiotics. The activity measured from strain co-transformed with the two empty plasmids was used as a negative control. Activities are presented as percentage relative to the negative control. All results are the average of at least three independent biological repeats. Error bars represent the standard deviation. Insert: Topology of MalE -DctA Helix 8b fusion relative to the cytoplasmic membrane^19^.

We have presented both *in vivo* and *in vitro* evidence that shows that DauA interacts with DctA. To identify the domains of both proteins involved in the interaction we exploited the BTH system. It is known that Slc26a proteins interact with various partners mainly via their STAS domain in plants, animals and bacteria^23,24^. To test the potential involvement of DauA STAS domain in the interaction with DctA we generated plasmid constructs expressing a truncated DauA lacking the STAS domain (DauA_1–444_) and plasmid constructs expressing only the STAS domain fused to the T18 or the T25 domain of adenylate cyclase. In *E*. *coli*, it has been shown the DctA interact with DcuS via its cytoplasmic amphipatic helix 8b (TM8)^19^. To test whether helix 8b is involved in complex formation between DctA and DauA, we expressed the TM8 of DctA fused at the N-terminus with MalE, and the T25 fragment on the C-terminus (Fig. 6). MalE is involved in maltose uptake and only active in the periplasm. When this construct was expressed, it was able to confer growth on maltose to a *malE* mutant indicating that the fusion to MalE is located in the periplasmic. β-galactosidase activity indicated a clear interaction of DauA _(1–444)_ with DctA. Furthermore, no interaction was detected between DauA and helix 8b of DctA. Finally, the isolated STAS domain of DauA does not interact with DctA (Fig. 6). These results suggest that DauA and DctA interact primarily via their transmembrane domain.

## Discussion

### Interconnected functionality in bacteria physiology

The results presented in this study underpin a fundamental question in bacterial physiology; how microbes integrate various physiological inputs in order to maximize nutrient utilization, cell growth to rapidly adapt to their environment. In this context, an increasing number of reports have confirmed the existence of co-regulation between various metabolic pathways in bacteria^25–27^. The consequences of these co-regulatory mechanisms is to preserve a metabolic equilibrium by adjusting central metabolism to the challenge produced by changes to nutrient source, starvation and/or stress. Metabolic co-regulation represents a safety net that allows rapid reprogramming of metabolism to facilitate cell adaptation. Functional studies on bacterial DauA have identified various carboxylic acids, bicarbonate and sulfate as substrates, highlighting the multifunctional roles of bacterial Slc26A transport systems^4–6,24,28^. Also, it is now well documented that the Slc26A transporters may initiate multiple interactions with different partners^8,29,30^. Hence, investigations aimed at identifying other potential DauA interacting partners, as well as investigating the interactome of bacterial Slc26A transporters may lead to the discovery of new important sensory and regulation mechanisms and reveal how these transporters participate in multiple cellular functions and potentially initiate metabolic co-regulation^8^. In *E*. *coli*, the co-purification and co-crystallization of the DauA STAS domain with the acyl-carrier protein suggest a role for DauA in fatty acid metabolism^24^. Hence, our data point toward a novel mechanism of metabolic co-regulation linking fatty acid biosynthesis and C_4_-diC acids metabolism. The interdependence of C_4_-diC metabolism via the TCA cycle and fatty acid biosynthesis have been shown in a range of bacteria and results in adaptation of membrane structure to temperature stress^31,32^. In this context it is noteworthy to note that in *Bacillus subtilis*, the stressosomes complex involved in the response to various stress (temperature, pH, osmolarity) is made of multiple copies of STAS domain containing proteins^15^.

### Model for DauA/DctA interaction

**Figure 7:**
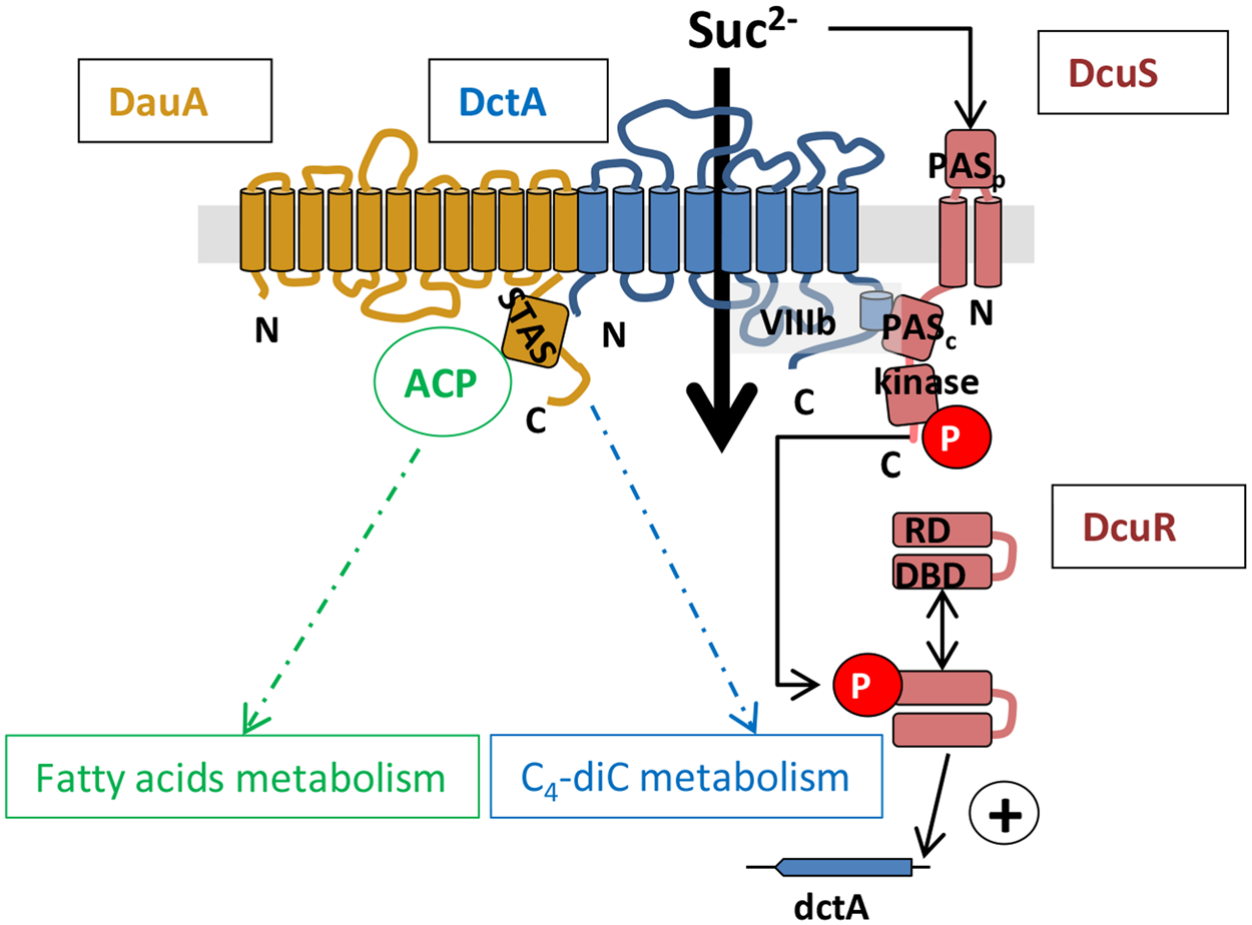
Model of the DauA/DctA complex function and integration of DauA in fatty acids and C_4_-diC metabolism. ACP: Acyl Carrier Protein, RD: regulator domain, DBD: DNA Binding Domain.

Based on our results, we propose a model for the function of DauA (Fig. 7).

Firstly, we have clearly shown, by phenotypic investigation, transport assay and western blot analysis that, when *E*. *coli* was grown at pH7 in minimal medium containing fumarate as sole carbon source, DauA was essential for optimal DctA function/expression. We have previously shown that, under our experimental conditions, the production of DctA dependent on the DcuS/DcuR two component system^4^.

Secondly, realization of the physical and functional interactions between bacterial C_4_-diC sensor kinases and transporters as accessory proteins has emerged in recent years and appears to be a common feature: in *E*. *coli*, under aerobic conditions, DctA forms a permanent complex with DcuS^19^. The role of DctA is to confer a fumarate-sensing function on DcuS (DcuS being constitutively “on” in the absence of DctA), hence the concept of an “sensitivity switch” for DctA^20^. Under anaerobic conditions, the C_4_-diC transporter DcuB takes over the role of DctA and forms a functional complex with DcuS^21^.

Taking into account the above evidence, our first hypothesis was that, under our experimental conditions, DauA may form a complex with DcuS. Our results using the bacterial two-hybrid analysis are in line with this hypothesis, and indicate that DauA forms a complex with DcuS and/or DctA. In order to dissect the hierarchy of this complex, we used co-purification and mass spectrometry analysis, which showed to our surprise that DauA interacts with DctA but not DcuS. This has been clearly confirmed by pull-down experiments and western blot analysis. Taken together, these results prove that DauA/DctA form a complex at the membrane. Whether DauA/DctA and DcuS form a tripartite complex at the membrane or the impact of DauA on DctA production occurs only via the DauA/DctA interaction remains to be determined. However, it has been shown that i) DctA and DcuS form a permanent complex, independently of their exposure to C_4_-diC^19^, ii) the function of DctA in converting DcuS to the sensory active state is independent of its transport activity, that (iii) the site for perceiving the C_4_-diC as the stimulus resides in DcuS^19,20^, and iv) under our experimental condition, DctA production depends totally on the presence of DcuS^4^. Therefore it is unlikely that the effect of DauA is due to the modulation of DctA activity, but may rather impact the “switch” function of DctA within the DctA/DcuS complex (Fig. 7). Finally, DauA appears to be inactive at pH7, hence its function as a sensor is independent of its transport activity. There is an increasing number of bacterial sensors that require an uptake system for normal function and sensing in bacteria. In these system, the uptake system can act as activity switch, positive or negative reporter of transporter flux or a signaling hub for the integration of multiple input (for review see^33,34^). In *E*. *coli*, the phosphate ABC transporter PtsSCAB_2_ interacts with the two-component system PhoR/B for phosphate sensing^35^, the hexose phosphate transporter UhpC interact with the sensor kinase UhpB^36^, the carboxylic acids transporter DctA and DcuB interact with DcuS^37^. Interestingly, in all these system, the sensing can by perceived by the transporter (PtsSCAB2) or the sensor kinase (DctA/DcuB), but the uptake process is dissociated from the sensor function of the associated transporter.

### Interaction of Slc26A protein with other secondary transporters

The STAS domains are the central structural features of the C-terminal cytoplasmic domains of Slc26 anion transport proteins. So far, all the interactions between SLC26A/SulP transporters and other proteins that have been identified in humans, plants and bacteria involved the STAS domain^8,23^. We have shown here via BTH analysis that the DauA/DctA complex interaction is between DauA and DctA transmembrane domains of the two proteins uncovering a potentially novel mechanism of integrating physiological signals (Fig. 7). Investigations aiming to identify precisely the interacting interfaces between DauA and DctA are needed. However, the interaction of Slc26A/SulP transporters with proteins localized in the membrane in humans, plants and bacteria seems to be the rule rather than the exception^8,24,38^. Particularly relevant, the direct interaction of various human Slc26A uptake systems with other transporters is now very well documented. Firstly, the existence of physical and functional interactions between the cystic fibrosis transmembrane conductance regulator (CFTR) Cl^−^ channel and several members of the SLC26 family (*i*.*e*. SLC26A3, A4, A5, A6, A8 and A9) has been established^8,29,30^. These interactions induce a reciprocal and unidirectional mechanism of regulation: stimulation or inhibition have been reported. Secondly, biochemical and functional analyses reveal interaction between SLC26A6/A3 and the citrate transporter NaDC-1^39^. The interaction results in mutual and reciprocal regulation which suggests a senso-regulatory molecular mechanism for oxalate/citrate homeostasis. This is a remarkable example of the interaction and co-regulation of carboxylic acid transporters^39^. Having the above in mind, it is quite tempting to speculate that bacterial SLC26A uptake systems, amongst which DauA, might have similar roles. The relationship between DauA/DctA complex formation and the presence of C4-diC, as well as whether the transporters regulate each others activity remains unclear and needs further investigation.

## Methods

### Bacterial strains and growth conditions

The strains and plasmids used in this study are listed in Table 2.

**Table 1:** Potential DauA interacting partner identified by nLS-MS-MS.

Gene	Description	Unique peptides detected	Seq. covered %
*atpF*	ATP synthase subunit b	2–3	16–23
*cls*	Cardiolipin synthase (CL synthase) (EC 2.7.8.-)	2–6	5–15
*dauA*	C4-dicarboxylic acid transporter DauA	5–12	7–19
*dctA*	Aerobic C_4_-dicarboxylate transport protein	2–4	8–25
*fruA*	PTS system fructose-specific EIIBC component (EIIBC-Fru)	1	3
*hflD*	High frequency lysogenization protein HflD	1–5	7–27
*lptB*	Lipopolysaccharide export system ATP-binding protein LptB (EC 3.6.3.-)	1–5	8–32
*nikE*	Nickel import ATP-binding protein NikE (EC 3.6.3.24)	1–3	5–14
*ybhL*	Inner membrane protein YbhL	1	3
*ycaR*	UPF0434 protein YcaR	1–2	35
*zraS*	Sensor protein ZraS (EC 2.7.13.3)	1–2	6

*E. coli* was routinely cultivated on Luria-Bertani broth (LB), LB-agar^40^, M9 minimal medium^41^ or enriched M9 (eM9) medium^42^. Ampicillin 100 μg/ml, Kanamycin 50 μg/ml and Chloramphenicol 30 μg/ml were used when required.

**Table 2:** Strains and plasmid use in this work.

Strains/ Plasmids	Genotype	Source
*E*. *coli* K-12		
WT (BW25113)	F-, Δ(*araD-araB*)567, Δ*lacZ4787*(::*rrnB-3*), λ-, *rph-1*, Δ(*rhaD-rhaB*)568, *hsdR514*	44
EK1	BW25113, but Δ*dauA*	4
EK2	BW25113, but Δ*dctA*	4
EK3	BW25113, but Δ*dauA/*Δ*dctA*	4
EK4	BW25113 encoding DauA-His^1^	4
EK5	EK2 encoding DauA-His^1^	4
EK6	BW25113 encoding Dcta-His^1^	4
EK7	EK1 encoding DctA-His^1^	4
EK9 IMW385	EK4 encoding DctA-FLAG^1^ MC4100, but λ[Φ(*dctA–lacZ*)hyb Amp^R^]	This study^45^
EK8	IMW385, but Δ*dauA*	4
**Plasmid**		
pBluescript-II KS+	Amp^R^	Stratagene
pMAK705	pSC101 derivative- rep(ts) - Cm^R^	43
pMW2031	*yfp(A206K)-dauA* with N-terminal His6-Tag, pBAD30 derivative (Amp^R^)	

Strain EK9 was constructed as follows: a fragment corresponding to the the last 501-base pairs (bp) of dctA containing the FLAG tag before the termination codon and a fragment correpomding to the 500 bp sequence downstream of *dctA* were amplified by PCR and cloned simultaneously into pBluescript (Stratagene). The *dctA*(FLAG)-tagged was subcloned into pMAK705 and transferred into the chromosome of EK4 as described^43^.

### Growth experiments

*E*. *coli* grew overnight in eM9 medium, washed twice in eM9 (carbon source free) at pH 7 and diluted to an OD_600_ of 0.05 in eM9 medium containing the appropriate carbon source at the appropriate pH. Growth was monitored for 24 hrs using a Synergy 2 platereader (Biotek).

### β-galactosidase assays

*E*. *coli* strains were cultured overnight in M9 minimal medium containing glucose as sole carbon source. The next day the cells were washed twice in carbon free M9 and diluted to an OD_600_ of 0.6 in succinate-M9. After 6 hrs, cells were harvested for measurement of β-galactosidase activity. β-galactosidase activity was measure according to Karinou *et al*.^4^

### Subcellular Fractionation

Cells were grown as for the β*-*galactosidase assays and fractionated according to procedures described previously^46^. The *DC protein assay* kit (Bio-Rad Laboratories) was used to measure protein concentration using bovine serum albumin as the standard.

### Western immunoblotting

Anti-His antibody (Qiagen); anti-FLAG antibody (Sigma), rabbit polyclonal anti-TatC antibody (kind gift of T. Palmer) polyclonal rabbit anti-DcuS (G. Unden, unpublished) were use to detect the proteins and the Western blotting was performed as described previously^46^. Proteins were detected using one of the following antibodies: anti-His antibody (Qiagen); anti- FLAG antibody (Sigma), rabbit polyclonal anti-TatC antibody raised against purified *E*. *coli* TatC protein (kind gift of T. Palmer) polyclonal rabbit anti-DcuS raised against purified E. coli DcuS (G. Unden, unpublished).

### Bacterial two-hybrid (BTH) system

This genetic screening is based on the reconstitution, in an *E*. *coli cyaA* ^*-*^ strain (deficient in endogenous adenylate cyclase), of a signal transduction pathway that takes advantage of the positive control exerted by cAMP. Two putative interacting proteins are genetically fused to two complementary fragments, T25 and T18, which constitute the catalytic domain of *Bordetella pertussis* adenylate cyclase. Association of the two-hybrid proteins results in functional complementation between T25 and T18 fragments and leads to cAMP synthesis. Cyclic AMP then triggers transcriptional activation of catabolic operons, such as lactose or maltose. *E*. *coli cyaA* ^*−*^ are unable to ferment maltose: they form white colonies on MacConkey indicator media containing maltose, while *cyaA* ^+^ bacteria form red colonies on the same media (the fermentation of maltose results in the acidification of the medium which is revealed by the colour change of the dye phenol red)^22^.

Plasmids harboring fusion proteins to T18 and T25 were co-transformed in *E*. *coli cyaA* ^*−*^ strain BTH101 and plated onto MacConkey medium supplemented with maltose, and cultured at 30 °C for 48 h. Red colonies signified possible interactions. An average colony was inoculated in 5 ml LB medium supplemented with appropriate antibiotics, and grown overnight at 30 °C. The next day potential interactions were quantified measuring β-galactosidase activity.

### Fluorescence microscopy

For the expression of mYFP-DauA plasmid pMW2031 encoding a fusion of His6-mYFP (monomeric YFP(A206) variant) to the N-terminus of DauA (His6-mYFP-DauA) was transformed into strain EK1. The cells EK4 or EK1pMW2031 were grown at pH 5 in M9 medium (Miller 1992) with 50 mM Na-succinate and arabinose (as indicated) to OD_578_ of 0.8. The membrane fraction (Janausch *et al*. 2002) (10 to 30 µg of protein) was subjected to SDS PAGE and DauA-His6 or His6-mYFP-DauA were detected in the Western blot by antibodies against the His6-tag (monoclonal first antibody (H-3), Santa Cruz) and m-IgGκ BP-HRP (second antibody, Santa Cruz) as the second antibody. For microscopy the bacteria were grown aerobically at 30 °C in LB medium (Miller 1992) supplemented with ampicillin (100 µg/ml). Overnight cultures were diluted 1/50 and grown to mid-exponential growth phase (OD_578_ 0.8). Then the bacteria were induced by 0, or 10 or 50 µM L-arabinose for one hour. 5 µl of the culture was washed and resuspended in 1x PBS buffer. The cells were fixed on microscope slides that were freshly coated with a thin layer of 1% agarose and covered with a coverslip. *In vivo* fluorescence microscopy was conducted with a Keyence Biozero BZ-8000 microscope.

### *In vivo* [^14^C]-fumarate transport assays

*In vivo* [^14^C]-fumarate transport assays were adapted from^47^. Briefly, cells were cultured in the same way as for the β*-*galactosidase assays using the carbon source and pH indicated. After 6 hrs, cells were harvested, washed twice in M9 minimal medium without carbon source and resuspended in the same medium to give a final OD 600 nm of 0.6. At zero time [^14^C]-fumarate (55 mCi/mmol). Samples of 300 μl were taken at 0, 1, 2, 3, 4, 5 and 6 min, and uptake was terminated by filtration through nitrocellulose filters (Millipore type HA; 0.45 μm pore size) under a constant vacuum. The radioactivity present on the filter was determined using a scintillation counter. Data were calibrated by using internal standards spotted on filters and counted in the same experiment.

### Purification of DauA for mass spectrometry analysis

EK2 cells were grown overnight in 3 L LB medium supplemented with fumarate for the induction of DctA. The next day the cells were pelleted by centrifugation, resuspended in lysis buffer A (Tris 50 mM, pH7.6, 100 mM NaCl, 0.05 M EDTA) and lysed with a constant cell disrupter (three passes at 20 kpsi). Cell debris was removed by centrifugation at 20,000 g for 45 min and the supernatant was further centrifuged at 20.000 g for 2 h. The pelleted membrane fraction was resuspended and membranes proteins were extracted with 2% DDM for 1 h at 4 °C and loaded onto a Co^2+^-affinity column (HisTrap, GE Healthcare). The protein was purified using an FPLC (AKTA purifier -GE Healthcare).

### DauA/DctA pull down

EK9 cells were grown and the membrane proteins were extracted as described above. Solubilized membrane proteins were incubated with cobalt coated sepharose beads and incubated for 1 h at 4 °C. DauA-His was purified by immobilized metal ion affinity chromatography (IMAC) and eluted using 500 mM imidazole. The elution fractions were further purified using Anti-FLAG^®^ M2 affinity Gel (Sigma) according to the manufacturer’s instructions and protein was eluted using 0.1 M glycine HCl pH 3.

### Data Availability

The data generated during and/or analysed during the current study are available from the corresponding author on reasonable request.

## Electronic supplementary material

Supplementary Information
