# Supplementary material for: The E. coli dicarboxylic acid transporters DauA act as a signal transducer by interacting with the DctA uptake system
Source: Sci Rep. 2017 Nov 27;7:16331. doi: 10.1038/s41598-017-16578-w (PMC5703999; doi:10.1038/s41598-017-16578-w)
Supplement: Supplementary file 1 — Supplementary Information [file 41598_2017_16578_MOESM1_ESM.pdf]

The *E. coli* dicarboxylic acid transporters DauA act as a signal transducer by interacting with the DctA uptake system

Eleni Karinou[1, $], Alexander Strecker[2], Paul A. Hoskisson[3], Gottfried Unden[2] and Arnaud Javelle[1,3,*]

[1]Division of Molecular Microbiology, College of Life Sciences, University of Dundee, Dundee, DD1 5EH, UK. [2]Institute for Microbiology and Wine Research, Johannes Gutenberg-University, Mainz, Germany. [3]Strathclyde Institute of Pharmacy and Biomedical Sciences, University of Strathclyde, 161 Cathedral Street, Glasgow, G4 0RE, UK.

[*] **Corresponding author:** Email: arnaud.javelle@strath.ac.uk

Phone: +44 141 548 3827

[$] **Present address:** Section of Microbiology and MRC Centre for Molecular Bacteriology and Infection, Imperial College London, London, United Kingdom

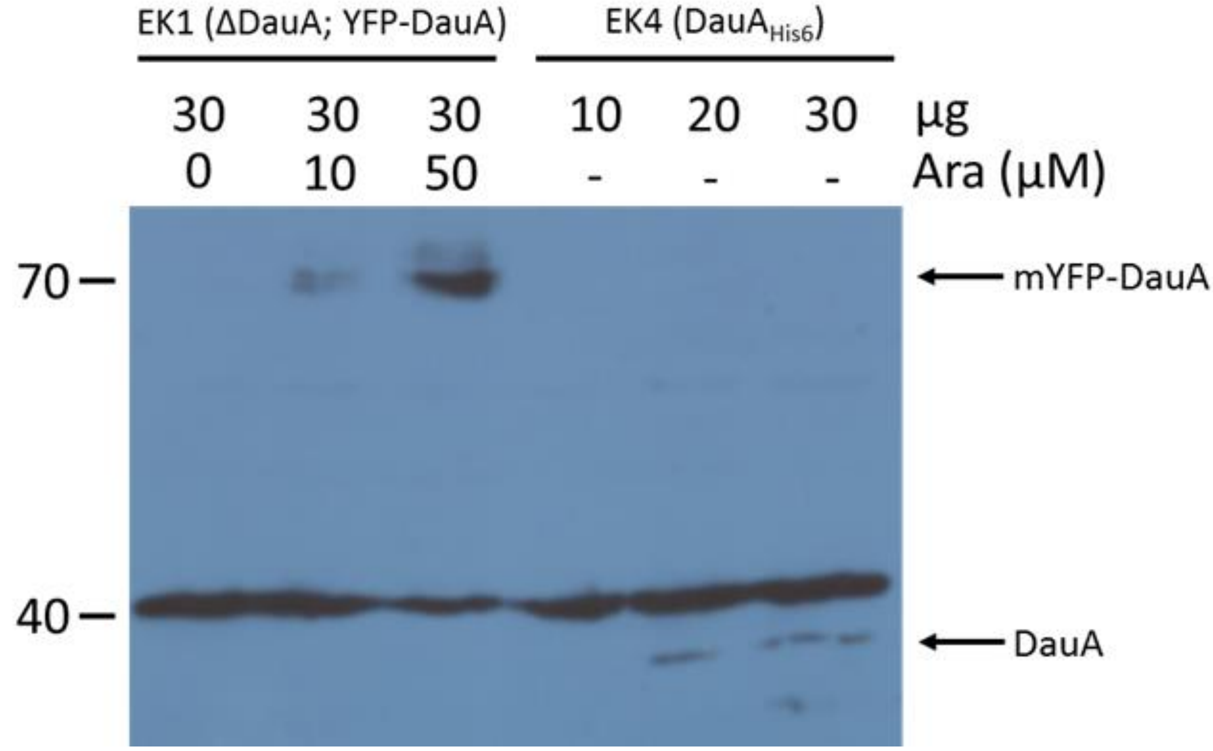

Figure S1. SDS-PAGE and Western blot analysis of strain EK4 (chromosomal *dauA-his6*) and strain EK1 with plasmid encoded *his6-mYFP-dauA* in a chromosomal *ΔdauA* background. Strain EK1 was grown with 0 to 50 µM arabinose. Membrane preparations of the bacteria were applied to SDS-PAGE (10 to 30 µg protein) and tested by Western blotting for the presence of DauA-His6 (Mr 35 kDa) or His6-mYFP-DauA (Mr 70 kDa). The left hand side on the gel shows the positions of protein standards (70 kDa and 40 kDa).

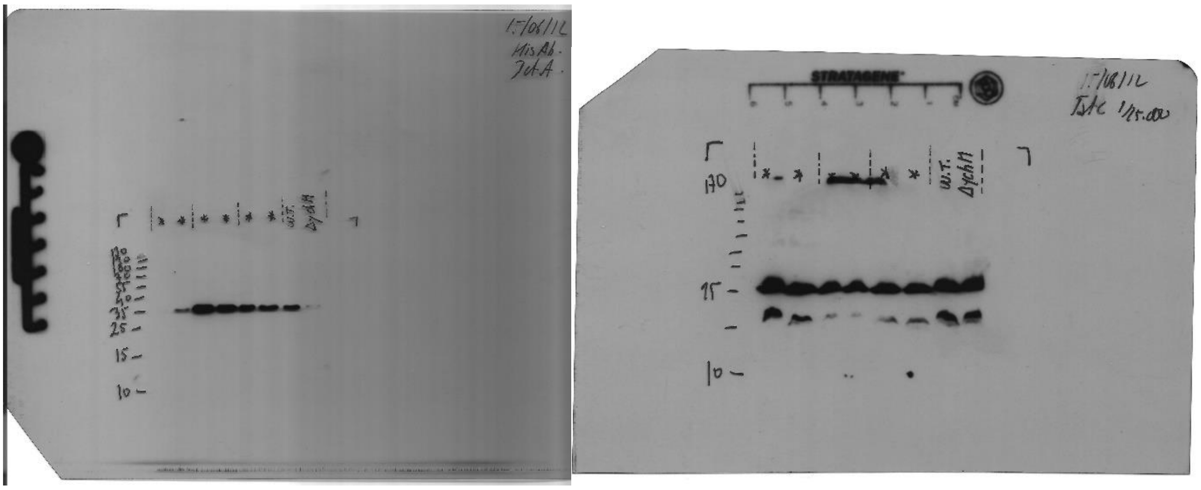

Original blot use to prepare the Figure 2 "The production of DctA depend on the presence on DauA". Left panel DctA-H6 detection. Right panel is the TatC loading control (25 kDa), the signal at 15 kDa is

a non-specific cross reaction in the strain BW25113 (See Cléon F, et al., 2015, *Mol. Microbiol.* 201: 98:111-29). Lanes 1-6 marked * are not related to the present study, labelling have been removed. Lane 7: DctA-H$_6$, Lane 8: DctA-H$_6$$\Delta$*dauA*. No modification of the contrast/brightness are apply. Images have been scanned using a HP Deskjet 2540 printer/scanner. These experiments have been done by Dr. Arnaud Javelle.

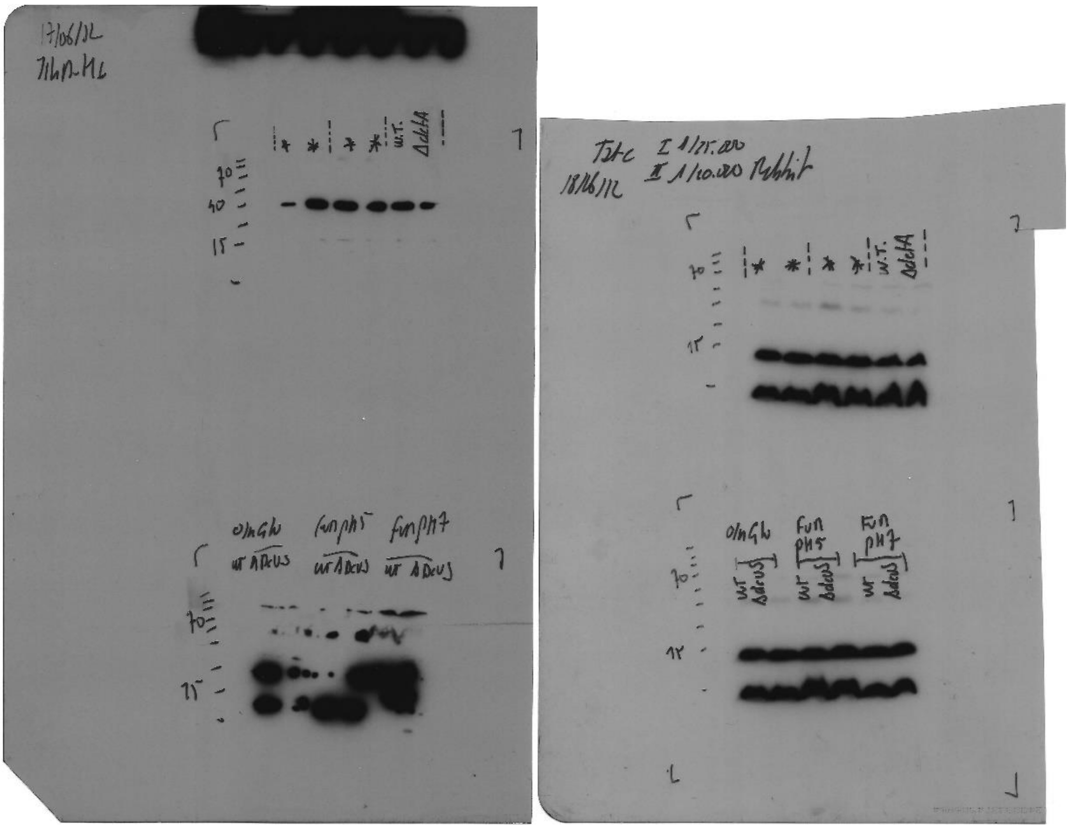

Original blot use to prepare the Figure 2 "The production of DctA depend on the presence on DauA". Left panel DauA-H6 detection, right panel is the TatC loading control (25 kDa), the signal at 15 kDa is a non-specific cross reaction in the strain BW25113 (See Cléon F, et al., 2015, *Mol. Microbiol.* 201: 98:111-29). Lanes 1-4 marked * are not related to the present study, labelling have been removed.. The bottom blot is not related to the present study. Lane 5: DauA-H$_6$, Lane 6: DauA-H$_6$$\Delta$*dctA*. No modification of the contrast/brightness are apply. Images have been scanned using a HP Deskjet 2540 printer/scanner. This experiments have been done by Dr. Arnaud Javelle.

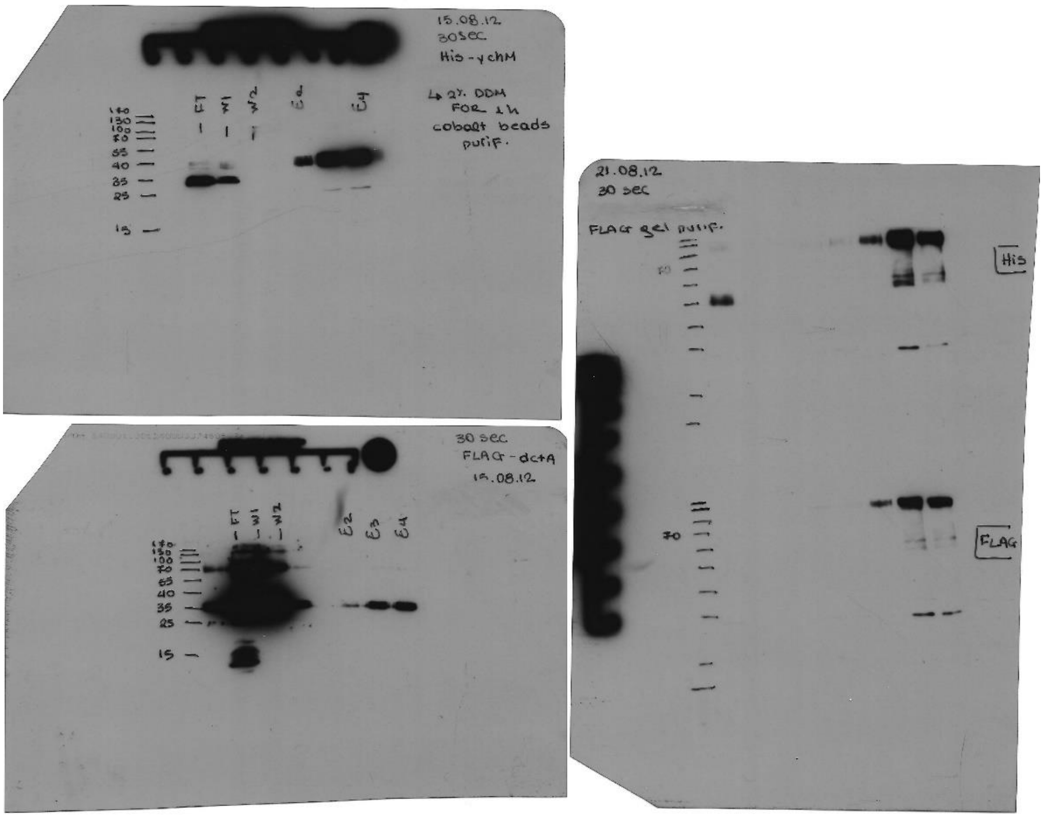

Original blot use to prepare the Figure 4 "DauA and DctA form a complex".

Left panels correspond to the first affinity (IMAC) purification of the DauA/DctA complex. Top, DauA-His is detected, bottom, the same membrane have been stripped and DauA-FLAG is detected. Only the elution fraction E2-E4 are presented in the manuscript.

Right panel: Fraction E1-E4 of the first IMAC have been polled and re-purified by FLAG-affinity. Top panel; DauA-His is detected; botton, DctA-FLAG is detected. Only the elution fraction E2-E4 are presented in the manuscript. Both blots have been prepared in parallel.

No modification of the contrast/brightness are apply. Images have been scanned using a HP Deskjet 2540 printer/scanner. These experiments have been done by Dr. Eleni Karinou.
